# Recent Advances and Clinical Outcomes of Kidney Transplantation

**DOI:** 10.3390/jcm9041193

**Published:** 2020-04-22

**Authors:** Charat Thongprayoon, Panupong Hansrivijit, Napat Leeaphorn, Prakrati Acharya, Aldo Torres-Ortiz, Wisit Kaewput, Karthik Kovvuru, Swetha R. Kanduri, Tarun Bathini, Wisit Cheungpasitporn

**Affiliations:** 1 Division of Nephrology, Department of Medicine, Mayo Clinic, Rochester, MN 55905, USA; charat.thongprayoon@gmail.com; 2 Department of Internal Medicine, University of Pittsburgh Medical Center Pinnacle, Harrisburg, PA 17105, USA; hansrivijitp@upmc.edu; 3 Department of Nephrology, Department of Medicine, Saint Luke’s Health System, Kansas City, MO 64111, USA; napat.leeaphorn@gmail.com; 4 Division of Nephrology, Department of Medicine, Texas Tech University Health Sciences Center, El Paso, TX 79905, USA; prakrati.c.acharya@gmail.com; 5 Department of Medicine, Ochsner Medical Center, New Orleans, LA 70121, USA; Aldo_t86@hotmail.com; 6 Department of Military and Community Medicine, Phramongkutklao College of Medicine, Bangkok 10400, Thailand; wisitnephro@gmail.com; 7 Division of Nephrology, Department of Medicine, University of Mississippi Medical Center, Jackson, MS 39216, USA; kkovvuru@umc.edu (K.K.); skanduri@umc.edu (S.R.K.); 8 Department of Internal Medicine, University of Arizona, Tucson, AZ 85724, USA; tarunjacobb@gmail.com

**Keywords:** kidney transplantation, renal transplantation, kidney transplant, renal transplant, transplant recipients, transplantation

## Abstract

Recent advances in surgical, immunosuppressive and monitoring protocols have led to the significant improvement of overall one-year kidney allograft outcomes. Nonetheless, there has not been a significant change in long-term kidney allograft outcomes. In fact, chronic and acute antibody-mediated rejection (ABMR) and non-immunological complications following kidney transplantation, including multiple incidences of primary kidney disease, as well as complications such as cardiovascular diseases, infections, and malignancy are the major factors that have contributed to the failure of kidney allografts. The use of molecular techniques to enhance histological diagnostics and noninvasive surveillance are what the latest studies in the field of clinical kidney transplant seem to mainly focus upon. Increasingly innovative approaches are being used to discover immunosuppressive methods to overcome critical sensitization, prevent the development of anti-human leukocyte antigen (HLA) antibodies, treat chronic active ABMR, and reduce non-immunological complications following kidney transplantation, such as the recurrence of primary kidney disease and other complications, such as cardiovascular diseases, infections, and malignancy. In the present era of utilizing electronic health records (EHRs), it is strongly believed that big data and artificial intelligence will reshape the research done on kidney transplantation in the near future. In addition, the utilization of telemedicine is increasing, providing benefits such as reaching out to kidney transplant patients in remote areas and helping to make scarce healthcare resources more accessible for kidney transplantation. In this article, we discuss the recent research developments in kidney transplants that may affect long-term allografts, as well as the survival of the patient. The latest developments in living kidney donation are also explored.

## 1. Introduction

Kidney transplantation is the optimal treatment for improving survival and quality of life for patients with end-stage kidney disease (ESKD) [1]. Advances in surgical, immunosuppressive and monitoring protocols have led to a significant improvement in overall one-year kidney allograft survival of >95% [2]. Nonetheless, there has not been a significant change in long-term kidney allograft outcomes. In fact, chronic and acute antibody-mediated rejection (ABMR) has continued to cause kidney allograft failures [3]. In addition, non-immunological complications following kidney transplantation, such as the recurrence of primary kidney disease and other complications, such as cardiovascular diseases, infections, and malignancy also play important roles in poor long-term allografts and patient survival [4,5,6].

In their research into immunologic monitoring and diagnostics in kidney transplants [7,8,9,10,11,12,13,14], a number of groups have made attempts in the recent past towards determining the peripheral molecular fingerprints of ongoing rejection [7,8] and predicting acute rejection [7]. Contemporary researchers have measured the levels of donor-derived cell-free DNA (dd-cfDNA) and showed higher predictive abilities for acute rejection [9,10,11,12], especially antibody-mediated rejection (ABMR) diagnostics in cases with a combination of donor specific antibodies (DSA) and dd-cfDNA [13,14]. In addition, a molecular microscope diagnostic system for the evaluation of allograft biopsies has been recently introduced within transplant practice, particularly in complex cases. This has mainly been introduced for the purpose of enhancing histological diagnostics [15].

Recent studies have been conducted aimed at preventing or treating ABMR [16,17]. In 2017, imlifidase (IdeS), an endopeptidase derived from Streptococcus pyogenes, was utilized in a desensitization regimen in an open-label phase 1–2 trial [16]. An instant impact was observed by a significant decline in plasma IgG levels. Another single-center phase 2 study that focused mainly on the pharmacokinetics, effectiveness and safety of IdeS treatment was conducted and proved a reduction in anti-human leukocyte antigen (HLA) antibodies using a complement-dependent cytotoxicity test [17].

In recent years, there has been significant progress in research into kidney transplantation and kidney donation [18,19,20,21,22,23,24,25,26,27,28,29,30,31,32,33,34,35,36,37,38,39,40,41,42,43,44,45,46,47,48,49,50,51,52,53,54,55,56,57,58,59,60,61,62,63,64,65,66,67,68,69,70,71,72,73,74,75,76,77,78,79,80,81,82,83,84], including articles [20,21,22,23,24,25,26,27,28,29,30,31,32,33,34,35,36,37,38,39,40,41,42,43,44,45,46,47,48,49,50,51,52,53,54,55,56,57,58,59,60] published in our current Special Issue "Recent Advances and Clinical Outcomes of Kidney Transplantation" (https://www.mdpi.com/journal/jcm/special_issues/outcomes_kidney_transplantation). 

In this article, we discuss the recent research developments in kidney transplantation that may impact long-term allografts and patient survival, as well as the latest developments in living kidney donation.

## 2. Non-HLA Antibodies in Transplantation

**Figure 1:**
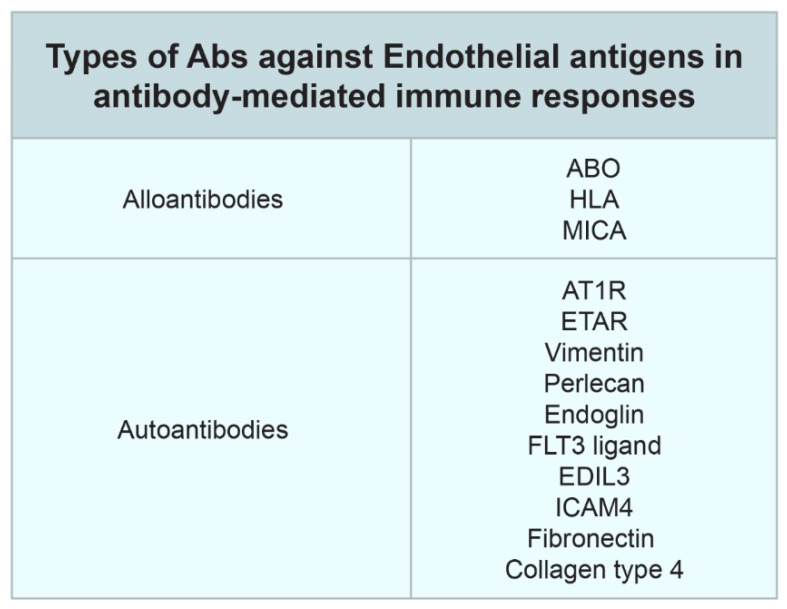
Post-transplant antibodies against human leukocyte antigen (HLA) and non-HLA antigens [68,78,86,87,88,89,90,91,92]. Abbreviations: human leukocyte antigen (HLA), major histocompatibility complex class I related chain A antigen (MICA); angiotensin type 1 receptor (AT1R); endothelin-1 type A receptor (Anti-ETAR); FMS-like tyrosine kinase 3 (FLT3); Epidermal growth factor-like repeats and discoidin I-like domain 3 (EDIL3); Intercellular adhesion molecule 4 (ICAM4).

When it comes to solid organ transplantation, one major immunological obstacle is the detection the non-self structures that exist in the donor cells. Human leukocyte antigens (HLA) are considered the most important non-self allo-antigens in organ transplantation. In addition, patients can form antibodies against targets other than HLA [85]. Multiple targets for these non-HLA antibodies have been studied in kidney transplantation over the last decade (Figure 1). Recent studies have provided findings that suggest the an importance of non-HLA mismatches between donors and recipients in the development of acute rejection and long-term kidney allograft outcomes [68,78,86,87,88,89,90,91,92]. 

## 3. Active AMR 

Chronic active ABMR is one of the major causes of long-term allograft loss [93,94,95]. Tocilizumab, a humanized monoclonal antibody targeting the interleukin (IL)-6 receptor, has been assessed in patients with acute and chronic active ABMR [96,97,98], given that IL-6 mediates various inflammatory and immunomodulatory pathways, including the expansion and activation of T cells and B cells [98]. Furthermore, there is a genetically engineered humanized Immunoglobulin (Ig)G1 monoclonal antibody that binds to IL-6, inhibiting its interaction with IL-6R. Direct inactivation of IL-6 may limit a rebound induced by the accumulation of IL-6 [99,100]. Preliminary investigations from phase 1–2 trials demonstrated the efficacy of the C1q inhibitor for the prevention of a delayed graft function (DGF) and to lessen the occurrence of chronic active ABMR [101,102]. Although the inhibition of the first step in both the classical and lectin pathways of complement activation may serve as another tool to overcome critical sensitization, such data need to be validated in larger cohorts. Several trials are currently being conducted, and new developments will conceivably provide us with practical ways to counteract the deleterious consequences of ABMR [103]. 

## 4. Cardiovascular Diseases in Kidney Transplant Recipients

**Figure 2:**
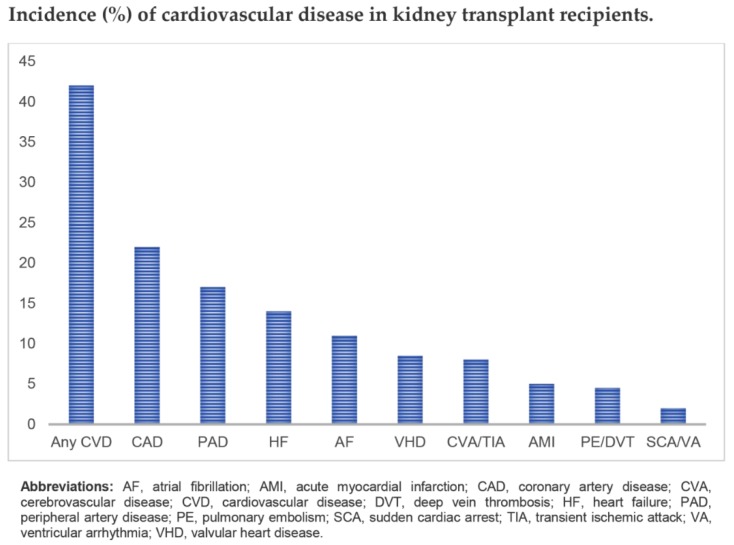
Incidence (%) of cardiovascular disease in kidney transplant recipients.

The burden of cardiovascular diseases on ESKD is improved after kidney transplantation [104]. However, it remains the leading cause of reduced early renal graft loss and mortality, as it is associated with significant morbidity and healthcare costs [104]. Major phenotypes of cardiovascular diseases among kidney transplant recipients include ischemic heart disease, congestive heart failure, valvular heart disease, arrhythmias and pulmonary hypertension (Figure 2). 

Reported risk factors for cardiovascular disease in kidney transplant recipients include inflammatory and immunosuppressive agents, episodes of allograft rejection, as well as traditional cardiovascular risk factors, such as hypertension, hyperlipidemia, smoking, obesity, chronic kidney disease, proteinuria, and diabetes mellitus, all of which add to a transplant recipient’s cardiovascular risk profile [104]. Hypertension is common among kidney transplant recipients and uncontrolled hypertension in kidney transplant recipients is associated with increased cardiovascular mortality and morbidity, and reduced allograft survival [105]. Furthermore, weight gain is also a significant problem in post-kidney transplant patients. Weight gain after transplantation can unfavorably affect patient outcomes [106]. Identifying these risk factors and adopting strategies to abolish these risk factors may potentially prevent, and help manage, post-transplant obesity. The underlying mechanisms for the increased occurrence of dyslipidemia post-transplant are due to immunosuppressive medications, proteinuria, and post-transplant diabetes [107,108].

The medical management of risk factors includes strategies employed in the chronic kidney disease (CKD) population, with credence given to approaches specific for kidney transplant recipients, such as the choice of maintenance immunosuppression, steroid tapering or withdrawal, and particular anti-hypertensive regimens (Table 1). Overall, cardiovascular morbidity and mortality in kidney transplant recipients has decreased over the last few decades, likely due to improved detection and the timely management of risk factors. Recognition of these complications is important in assessing cardiovascular disease risk in kidney transplant recipients, and optimizing screening and therapeutic approaches. These include lifestyle and immunosuppressive regimen modification, as well as the best feasible regimen for glycemic and lipid controls according to an individual’s metabolic profile and medical history.

## 5. Preexisting Diabetes and Post-Transplantation Diabetes 

Preexisting diabetes and post-transplantation diabetes confer reduced patient and graft survival in kidney transplant recipients [71,73,125]. Hyperglycemia is present in nearly 90% of kidney transplant recipients in the immediate postoperative period, but it is not sustained in the majority [126]. In addition to the general risk factors for diabetes, there are also certain transplantation-related factors (e.g., specific immunosuppressive agents, surgical stress and inflammation, nutritional interventions) placing kidney transplant recipients at elevated risk of hyperglycemia [126]. Some transplant immunosuppressive medications, including corticosteroids, calcineurin Inhibitors (CNIs), and mammalian target of rapamycin (mTOR) inhibitors, are associated with a higher incidence of metabolic complications such as post-transplantation diabetes. CNIs impair insulin secretion and sensitivity and directly damage pancreatic islet cells [127]. 

A robust evidence base guiding precise glycemic goals is currently lacking in kidney transplant recipients. Management is largely guided by evidence from the general diabetes population [71,73,125]. Hospital management of hyperglycemia is primarily achieved through an insulin regimen that takes into account rapid changes in glucocorticoid doses, nutritional modalities and renal function during the immediate post-transplantation period. There is an opportunity to use oral or non-insulin injectable agents in a considerable number of patients by the time they are discharged from the hospital, or in the long run. The use of specific oral or non-insulin injectable agents is guided by patient specifics and the pharmacologic properties of medications. Although several studies have suggested the safe use of sodium glucose transport 2 (SGLT2) inhibitors in kidney transplant recipients [128], future studies assessing their efficacy and safety are needed, since SGLT2 inhibitor treatment also carries an increased risk of genital tract infections and, possibly, of urinary tract infections [129]; kidney transplant recipients are particularly susceptible to infections due to immunosuppressive regimens. 

## 6. Posttransplant Malignancy

Cancer is one of the three major causes of death after kidney transplantation [130,131]. Posttransplant malignancy occurrence is widely recognized (Table 2). The effect of viral infections, induction and immunosuppressive maintenance regimens have been proposed as important risk factors for posttransplant malignancy. The increased risk of cancer may be due to viral reactivation induced by immunosuppressive agents or impaired immune surveillance leading to faster tumor growth [132]. A higher degree of immunosuppression is associated with an increased risk of malignancy, and calcineurin inhibitors can promote carcinogenesis [132].

## 7. Infection

Solid organ transplant recipients are at greater risk of infection than the non-immunosuppressed population (Table 3) [134]. Infections are the most common non-cardiovascular causes of mortality following kidney transplantation, accounting for 15%–20% of mortality [131,135]. The first six months post-transplant is the time of greatest infection risk. There are also times when patients encounter adverse reactions to immunosuppressive agents [136,137]. Among all infectious complications, viruses are considered to be the most common agents [138]. Herpes simplex virus, varicella zoster virus, BK polyomavirus, cytomegalovirus, Epstein–Barr virus, hepatitis B virus, and adenovirus are well-known etiologic agents of viral infections in kidney transplant patients worldwide [138]. In order to prevent opportunistic infections in kidney transplant recipients, antimicrobial prophylaxis is recommended after kidney transplantation. The recommended prophylactic method after transplant differs based on the organism, as well as individual patient characteristics.

## 8. Latest Developments in Living Kidney Donation

Living donor kidney transplants are the best option for many patients with ESKD for several reasons, including (1) better long-term graft survival, (2) no need to wait on the transplant waiting list for a kidney from a deceased donor, (3) transplant surgery can be planned and (4) lower risks of rejection and DGF [139]. Living donor kidney transplantation is the optimal treatment for patients with ESKD [139]. The expansion of living donor programs was made possible by new modes of living donation and by the extension of the living donor pool [139]. 

To expand the donor pool, a well-developed paired kidney donation program and the adequate reimbursement of costs associated with donation are fundamental elements [140]. Paired kidney donation provides living kidney donation for noncompatible donor/recipient pairs that otherwise would not be feasible or need desensitization [141]. Other possible approaches for increasing the donor pool include ABO-incompatible transplantation [142], the utilization of higher risk donors, advanced donation with a voucher system, and providing donors with financial incentives [141,143,144]. 

Over the past decade, the long-term risks of kidney donation have been described. Living donors seem to have a higher risk of ESKD, particularly in obese donors and also for African American donors with an apolipoprotein L1 (APOL1) high-risk genotype. In African American living kidney donors, those with the APOL1 high-risk genotype (prevalent in about 13% of African Americans in the United States) had an almost three times more accelerated decline in estimated glomerular filtration rate (eGFR) after adjusting for pre-donation eGFR than those with a low-risk genotype [145].

## 9. Post-Transplant Hyperparathyroidism and Bone Disease

**Figure 3:**
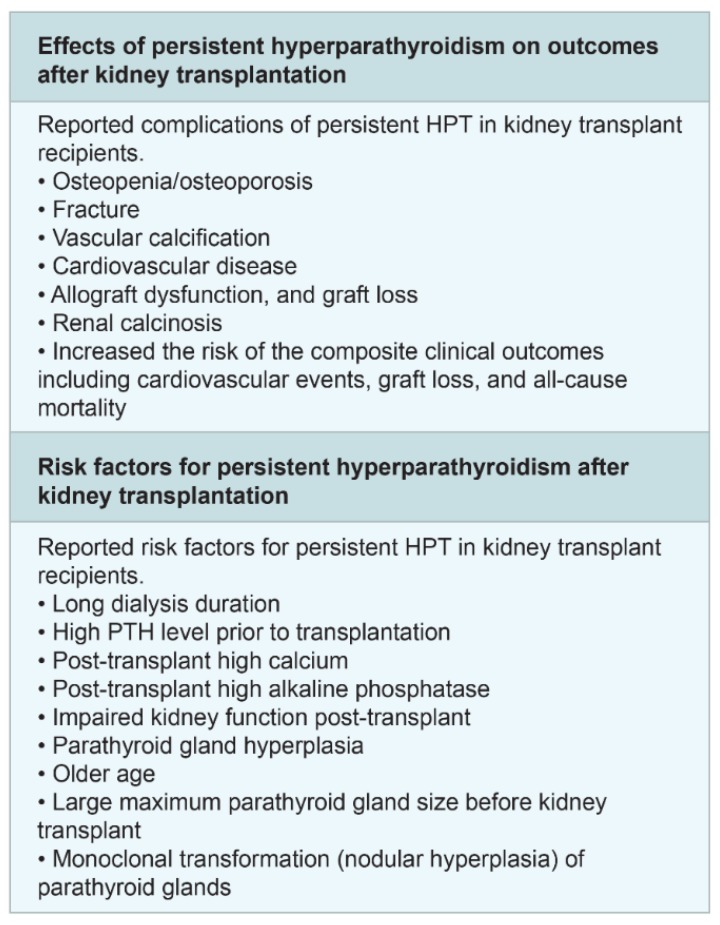
Effects and risk factors of post-transplant hyperparathyroidism.

Successful renal transplantation results in a reduction in parathyroid hormone (PTH), especially during the first 3 months after transplantation [146]. However, elevated PTH levels can still be found in 30% to 60% of patients 1 year after transplantation. Persistent hyperparathyroidism following kidney transplantation can result in notable complications, such as fracture/bone diseases, cardiovascular disease, vascular calcification, and allograft dysfunction (Figure 3). Associated factors for persistent hyperparathyroidism are long dialysis duration, high PTH levels prior to transplantation, lower eGFR post-transplant, post-transplant hypercalcemia, and post-transplant high alkaline phosphatase.

## 10. Potential Directions and Future Scope 

Researchers need to instantly shift their focus on the unaddressed concerns with respect to kidney transplants. Because of the limited supply of organs, numerous potential recipients still have to spend more time in dialysis, waiting for a transplant. Sensitization to HLA antigens inhibits the recipients’ access to transplants, compromising the survival of the graft due to chronic and acute AMR. The publication of complete data from a multi-center second-phase test that explores how IdeS is useful in desensitization is underway (NCT02790437). The phase 3 trial, uncovering the impact of clazakizumab following transplantation, was launched recently, with the outcomes of the phase 2 trial to be released soon. 

Moreover, the lack of experienced and skilled professionals could hinder the diagnostic correctness of complications following transplantation. Furthermore, medication non-adherence among patients could increase the alloimune reaction. Notably, medical research on the costimulation blockade during kidney transplantation is underway. A randomized sixty-month multi-center study (CIRRUS, NCT03663335) in kidney transplant is also underway, with the aim of defining the range of dosage and assessing the tolerability, safety, and effectiveness of some newly developed anti-CD40 monoclonal antibodies in two distinct cohorts in comparison to a tacrolimus-based regimen. Recently, a phase 2a clinical trial, with the purpose of assessing how effective the dual costimulation blockade with anti-CD40 (VIB4920) is when combined with belatacept in kidney transplantation patients (NCT04046549), was registered. 

Big data is increasingly being utilized, with the establishment of a large collection of cohorts and the usage of electronic health records (EHRs) in kidney transplantation and artificial intelligence, which might be useful in solving problems related to the survival analysis of patients who have gone through kidney transplantation [147,148,149,150,151,152,153,154,155]. In the present era, it is strongly believed that big data and artificial intelligence will greatly reshape the research done on kidney disease and, consequently, improve the general clinical practice of nephrology [156].

The benefits of telemedicine include reaching out to patients in remote areas and helping to make scarce healthcare resources more accessible. As telemedicine applications continue to proliferate, studies have demonstrated that telehealth for transplant care may be associated with a reduction in cost and time, and may also improve access to transplantation for ESKD patients [157,158].

## 11. Conclusions 

The most recent endeavors in kidney transplantation tend to mainly focus on noninvasive monitoring, as well as the improvement of histological diagnostics with the aid of molecular techniques. Such studies offer creative means that can be used to find immunosuppressive agents, which can effectively overcome critical sensitization, prevent the creation of anti-HLA antibodies, treat chronic active ABMR, and reduce non-immunological complications following kidney transplantation, such as the recurrence of primary kidney disease and other complications, such as cardiovascular diseases, infections, and malignancy. In the present era of utilizing EHRs, it is strongly believed that big data and artificial intelligence will reshape the research done on kidney transplantation in the near future. In addition, the utilization of telemedicine is increasing, providing benefits such as reaching out to kidney transplant patients in remote areas and helping to make scarce healthcare resources more accessible for kidney transplantation.

## Figures and Tables

**Table 1:** Cardiovascular risk factors among kidney transplant recipients and suggested management.

Cardiovascular Risk Factor	Suggested Management	Reference
**Traditional risk factors**		
Hypertension	Monitor each visit Target BP < 130/80 mmHg (ACC/AHA, 2017) Initial treatment with CCB ACEI/ARB if > 1 g/day proteinuria	[109,110,111,112]
Diabetes	Monitor for post-transplant DM annually Target HbA1c 7.0–7.5% (KDIGO, 2009) Low-dose ASA in all atherosclerotic CVD	[109,113]
Cigarette smoking	Screen annually Offer intervention for smoke cessation	[109,114]
Dyslipidemia	Monitor annually Use of statins favored in all KTx (KDIGO, 2014)	[109,115]
Obesity	Monitor BMI and weight circumference Healthy diet and exercise BMI target < 35 kg/m2	[109,116]
**Non-traditional risk factors**		
eGFR < 45 ml/min/1.73m2	Increased use of living donor organs if possible Check serum creatinine at least annually Avoid nephrotoxic medications	[109,117]
Proteinuria	ACEI/ARB if > 1 g/day proteinuria Check urine analysis at least annually	[109,118,119]
Left ventricular hypertrophy	Check ECG, echocardiography Treat underlying hypertension	[109,120]
Anemia	Treatment similar to CKD guidelines Check CBC	[109,121,122]
Acute rejection episodes	Treat rejections as per KDIGO, 2009	[109,123,124]

American College of Cardiology (ACC); angiotensin-converting enzyme inhibitor (ACEI); American Heart Association (AHA); angiotensin-II receptor blocker (ARB); aspirin (ASA); body mass index (BMI); blood pressure (BP); complete blood count (CBC); calcium-channel blockers (CCB); chronic kidney disease (CKD); cardiovascular disease (CVD); diabetes mellitus (DM); electrocardiography (ECG); estimated glomerular filtration rate (eGFR); Kidney Diseases Improving Global Outcomes (KDIGO); kidney transplant (KTx).

**Table 2:** Standardized incidence ratio of cancers in kidney transplant recipients [133].

Cancer	Standardized Incidence Ratio (95% CI)
Lip cancer	29.45 (17.85–48.59)
Non-melanoma skin cancer	12.14 (6.37–23.13)
Renal cell carcinoma	10.77 (6.40–18.12)
Non-Hodgkin lymphoma	10.66 (8.54–13.31)
Thyroid cancer	5.04 (3.79–6.71)
Hodgkin lymphoma	4.90 (3.09–7.78)
Urinary bladder cancer	3.52 (1.48–8.37)
Melanoma	2.48 (1.08–5.67)
Hepatocellular carcinoma	2.45 (1.63–3.66)
Gastric cancer	1.93 (1.60–2.34)
Colon cancer	1.85 (1.53–2.23)
Lung cancer	1.68 (1.29–2.19)
Ovarian cancer	1.60 (1.23–2.07)
Pancreatic cancer	1.53 (1.23–1.91)
Breast cancer	1.11 (1.11–1.24)

Confidence Interval (CI).

**Table 3:** Infection post kidney transplantation.

<1 Month	1–6 Month	>6 Month
Bacterial infection * UTI (mainly E Coli, Enterobacteriaceae, Pseudomonas, Enterococcus) Respiratory Catheter, drainage sites, wound, perinephric fluid collection, urinary stent infections Bacteremia C diff colitis	**Bacterial infection** With prophylaxis ** -C diff colitis, Mycobacterium species Without prophylaxis -Listeria, Nocardia	**Bacterial infection** UTI Pneumonia
		**Viral infection** CMV (colitis or retinitis) Hepatitis (B and C) EBV, HSV, HHV-8, papillomavirus (associated with malignancy) VZV, BK virus, parvovirus
	**Viral infection** With prophylaxis—BK, Adenovirus, Influenza, EBV, HCV, Parvovirus Without prophylaxis—HSV, CMV, VZV,	
**Viral infection** HSV Donor-derived—HIV, Hepatitis, CMV, BK, LCM virus, West Nile virus, Rabies		
		**Fungal infection** Cryptococcus, Rhodococcus, Aspergillus, pneumocystis, Mucor
	**Fungal infection** With prophylaxis—Aspergillus, Cryptococcus, Mucor Without prophylaxis—Pneumocystis jiroveci	
**Fungal infection** Candida (can be donor derived or pre—TX colonization)		
	**Parasitic infection** Toxoplasma, Strongyloides, T. cruzi, Leishmaniasis	
**Parasitic infection** Donor-derived—Malaria, Babesia, Balamuthia, T. cruzi		

* Center-dependent multidrug resistant bacteria like Methicillin-resistant Staphylococcus aureus (MRSA), Vancomycin-resistant Enterococcus (VRE), extended-spectrum beta-lactamases (ESBLs); ** With prophylaxis – with Bactrim and Gancyclovir/Valganciclovir; Abbreviations: cytomegalovirus (CMV), lymphocytic choriomeningitis virus (LCM), Epstein-Barr Virus (EBV), hepatitis B virus (HBV), hepatitis C virus (HCV), Trypanosoma cruzi (T. cruzi), Varicella Zoster virus (VZV), human herpes virus 8 (HHV-8).

## References

[1] Abecassis M., Bartlett S.T., Collins A.J., Davis C.L., Delmonico F.L., Friedewald J., Hays R., Howard A., Jones E., Leichtman A.B. (2008). Kidney transplantation as primary therapy for end-stage renal disease: A National Kidney Foundation/Kidney Disease Outcomes Quality Initiative (NKF/KDOQITM) conference. Clin. J. Am. Soc. Nephrol..

[2] Loupy A., Lefaucheur C., Vernerey D., Prugger C., Duong van Huyen J.P., Mooney N., Suberbielle C., Frémeaux-Bacchi V., Méjean A., Desgrandchamps F. (2013). Complement-binding anti-HLA antibodies and kidney-allograft survival. N. Engl. J. Med..

[3] Viklicky O., Novotny M., Hruba P. (2020). Future developments in kidney transplantation. Curr. Opin. Organ Transplant..

[4] Faenza A., Fuga G., Nardo B., Donati G., Cianciolo G., Scolari M., Stefoni S. (2007). Metabolic Syndrome After Kidney Transplantation. Transplant. Proc..

[5] Cohen-Bucay A., Gordon C.E., Francis J.M. (2019). Non-immunological complications following kidney transplantation. F1000Research.

[6] Gill J.S., Abichandani R., Kausz A.T., Pereira B.J. (2002). Mortality after kidney transplant failure: The impact of non-immunologic factors. Kidney Int..

[7] Roedder S., Sigdel T., Salomonis N., Hsieh S., Dai H., Bestard O., Metes D., Zeevi A., Gritsch A., Cheeseman J. (2014). The kSORT Assay to Detect Renal Transplant Patients at High Risk for Acute Rejection: Results of the Multicenter AART Study. PLoS Med..

[8] Garg N., Samaniego M.D., Clark D., Djamali A. (2017). Defining the phenotype of antibody-mediated rejection in kidney transplantation: Advances in diagnosis of antibody injury. Transplant. Rev..

[9] Bloom R.D., Bromberg J.S., Poggio E.D., Bunnapradist S., Langone A.J., Sood P., Matas A.J., Mehta S., Mannon R.B., Sharfuddin A. (2017). Cell-Free DNA and Active Rejection in Kidney Allografts. J. Am. Soc. Nephrol..

[10] Huang E., Sethi S., Peng A., Najjar R., Mirocha J., Haas M., Vo A., Jordan S.C. (2019). Early clinical experience using donor-derived cell-free DNA to detect rejection in kidney transplant recipients. Arab. Archaeol. Epigr..

[11] Gielis E.M., Ledeganck K.J., Dendooven A., Meysman P., Beirnaert C., Laukens K., De Schrijver J., Van Laecke S., Van Biesen W., Emonds M.-P. (2019). The use of plasma donor-derived, cell-free DNA to monitor acute rejection after kidney transplantation. Nephrol. Dial. Transplant..

[12] Oellerich M., Shipkova M., Asendorf T., Walson P.D., Schauerte V., Mettenmeyer N., Kabakchiev M., Hasche G., Gröne H., Friede T. (2019). Absolute quantification of donor-derived cell-free DNA as a marker of rejection and graft injury in kidney transplantation: Results from a prospective observational study. Arab. Archaeol. Epigr..

[13] Jordan S.C., Bunnapradist S., Bromberg J.S., Langone A.J., Hiller D., Yee J., Sninsky J.J., Woodward R., Matas A.J. (2018). Donor-derived Cell-free DNA Identifies Antibody-mediated Rejection in Donor Specific Antibody Positive Kidney Transplant Recipients. Transplant. Direct.

[14] Sigdel T., Archila F.A., Constantin T., Demko Z., Liberto J.M., Damm I., Towfighi P., Navarro S., Kirkizlar E., Demko Z. (2018). Optimizing Detection of Kidney Transplant Injury by Assessment of Donor-Derived Cell-Free DNA via Massively Multiplex PCR. J. Clin. Med..

[15] Reeve J., Böhmig G.A., Eskandary F., Einecke G., Gupta G., Madill-Thomsen K., Mackova M., Halloran P.F. (2019). INTERCOMEX MMDx-Kidney study group Generating automated kidney transplant biopsy reports combining molecular measurements with ensembles of machine learning classifiers. Arab. Archaeol. Epigr..

[16] Jordan S.C., Lorant T., Choi J. (2017). IgG Endopeptidase in Highly Sensitized Patients Undergoing Transplantation. N. Engl. J. Med..

[17] Lorant T., Bengtsson M., Eich T., Eriksson B.-M., Winstedt L., Järnum S., Stenberg Y., Robertson A.-K., Mosén K., Björck L. (2018). Safety, immunogenicity, pharmacokinetics, and efficacy of degradation of anti-HLA antibodies by IdeS (imlifidase) in chronic kidney disease patients. Arab. Archaeol. Epigr..

[18] Bray R.A., Gebel H.M., Townsend R., Roberts M.E., Polinsky M., Yang L., Meier-Kriesche H.-U., Larsen C.P. (2018). De novo donor-specific antibodies in belatacept-treated vs cyclosporine-treated kidney-transplant recipients: Post hoc analyses of the randomized phase III BENEFIT and BENEFIT-EXT studies. Arab. Archaeol. Epigr..

[19] Leibler C., Matignon M., Moktefi A., Samson C., Zarour A., Malard S., Boutin E., Pilon C., Salomon L., Natella P.-A. (2019). Belatacept in renal transplant recipient with mild immunologic risk factor: A pilot prospective study (BELACOR). Arab. Archaeol. Epigr..

[20] Kolonko A., Słabiak-Błaż N., Karkoszka H., Więcek A., Piecha G. (2020). The Preliminary Results of Bortezomib Used as A Primary Treatment for An Early Acute Antibody-Mediated Rejection after Kidney Transplantation—A Single-Center Case Series. J. Clin. Med..

[21] Knobbe T., Douwes R.M., Kremer D., Swarte J.C., Eisenga M., Gomes-Neto A.W., Van Londen M., Peters F., Blokzijl H., Nolte I.M. (2020). Altered Gut Microbial Fermentation and Colonization with Methanobrevibacter smithii in Renal Transplant Recipients. J. Clin. Med..

[22] Osté M.C.J., Flores-Guerrero J.L., Gruppen E.G., Kieneker L.M., Connelly M.A., Otvos J., Dullaart R.P., Bakker S.J.L. (2020). High Plasma Branched-Chain Amino Acids Are Associated with Higher Risk of Post-Transplant Diabetes Mellitus in Renal Transplant Recipients. J. Clin. Med..

[23] Deen C., Van Der Veen A., Gomes-Neto A.W., Geleijnse J.M., Berg K.J.B.-V.D., Heiner-Fokkema M., Kema I., Bakker S.J.L. (2020). Urinary Excretion of N1-methyl-2-pyridone-5-carboxamide and N1-methylnicotinamide in Renal Transplant Recipients and Donors. J. Clin. Med..

[24] Sotomayor C.G., Groothof D., Vodegel J.J., Gacitúa T.A., Gomes-Neto A.W., Osté M.C.J., Pol R.A., Ferreccio C., Berger S.P., Chong G. (2020). Circulating Arsenic is Associated with Long-Term Risk of Graft Failure in Kidney Transplant Recipients: A Prospective Cohort Study. J. Clin. Med..

[25] Byambasukh O., Osté M.C.J., Gomes-Neto A.W., Berg E.V.D., Navis G., Bakker S.J.L., Byambasukh O., Byambasukh O. (2020). Physical Activity and the Development of Post-Transplant Diabetes Mellitus, and Cardiovascular- and All-Cause Mortality in Renal Transplant Recipients. J. Clin. Med..

[26] Choi S., Lee K.W., Park J.B., Kim K., Jang H.-R., Huh W., Kang E.-S. (2020). C3d-Positive Preformed DSAs Tend to Persist and Result in a Higher Risk of AMR after Kidney Transplants. J. Clin. Med..

[27] Klont F., Kieneker L.M., Gomes-Neto A.W., Stam S.P., Hacken N.H.T., Kema I., Van Beek A.P., Berg E.V.D., Horvatovich P., Bischoff R. (2020). Female Specific Association of Low Insulin-Like Growth Factor 1 (IGF1) Levels with Increased Risk of Premature Mortality in Renal Transplant Recipients. J. Clin. Med..

[28] Flothow D., Suwelack B., Pavenstädt H., Schütte-Nütgen K., Reuter S. (2020). The Effect of Proton Pump Inhibitor Use on Renal Function in Kidney Transplanted Patients. J. Clin. Med..

[29] Bailey P.K., Caskey F.J., MacNeill S., Tomson C., Dor F., Ben-Shlomo Y. (2019). Beliefs of UK Transplant Recipients about Living Kidney Donation and Transplantation: Findings from a Multicentre Questionnaire-Based Case–Control Study. J. Clin. Med..

[30] Yang D., Thamcharoen N., Cardarelli F. (2019). Management of Immunosuppression in Kidney Transplant Recipients Who Develop Malignancy. J. Clin. Med..

[31] Douwes R.M., Gomes-Neto A.W., Schutten J., Berg E.V.D., De Borst M.H., Berger S.P., Touw D., Hak E., Blokzijl H., Navis G. (2019). Proton-Pump Inhibitors and Hypomagnesaemia in Kidney Transplant Recipients. J. Clin. Med..

[32] Tubben A., Sotomayor C.G., Post A., Minovic I., Frelink T., De Borst M.H., Said M.Y., Douwes R.M., van den Berg E., Rodrigo R. (2019). Urinary Oxalate Excretion and Long-Term Outcomes in Kidney Transplant Recipients. J. Clin. Med..

[33] Piyasiridej S., Townamchai N., Udomkarnjananun S., Vadcharavivad S., Pongpirul K., Wattanatorn S., Sirichindakul B., Avihingsanon Y., Tungsanga K., Eiam-Ong S. (2019). Plasmapheresis Reduces Mycophenolic Acid Concentration: A Study of Full AUC0-12 in Kidney Transplant Recipients. J. Clin. Med..

[34] Gacitúa T.A., Sotomayor C.G., Groothof D., Eisenga M., Pol R.A., De Borst M.H., Gans R.O.B., Berger S.P., Rodrigo R., Navis G. (2019). Plasma Vitamin C and Cancer Mortality in Kidney Transplant Recipients. J. Clin. Med..

[35] Deen C., Van Der Veen A., Van Faassen M., Minović I., Gomes-Neto A.W., Geleijnse J.M., Berg K.J.B.-V.D., Kema I., Bakker S.J.L. (2019). Urinary Excretion of N1-Methylnicotinamide, as a Biomarker of Niacin Status, and Mortality in Renal Transplant Recipients. J. Clin. Med..

[36] Katou S., Globke B., Morgul M., Vogel T., Struecker B., Otto N., Reutzel-Selke A., Marksteiner M., Brockmann J., Pascher A. (2019). Urinary Biomarkers α-GST and π-GST for Evaluation and Monitoring in Living and Deceased Donor Kidney Grafts. J. Clin. Med..

[37] Hwang H., Hong K.-W., Kim J., Kim Y., Moon J., Jeong K., Lee S.-H. (2019). The Korean Organ Transplantation Registry Study Group; Korean Organ Transplantation Registry Study Group Validation of Identified Susceptible Gene Variants for New-Onset Diabetes in Renal Transplant Recipients. J. Clin. Med..

[38] Yepes-Calderón M., Sotomayor C.G., Kretzler M., Gans R.O.B., Berger S.P., Navis G., Ju W., Bakker S.J.L. (2019). Urinary Epidermal Growth Factor/Creatinine Ratio and Graft Failure in Renal Transplant Recipients: A Prospective Cohort Study. J. Clin. Med..

[39] Maxeiner A., Bichmann A., Oberländer N., El-Bandar N., Sugünes N., Ralla B., Biernath N., Liefeldt L., Budde K., Giessing M. (2019). Native Nephrectomy before and after Renal Transplantation in Patients with Autosomal Dominant Polycystic Kidney Disease (ADPKD). J. Clin. Med..

[40] Nieuwenhuijs-Moeke G.J., Huijink T.M., Pol R.A., El Moumni M., Burgerhof J.G., Struys M., Berger S.P. (2019). Intraoperative Fluid Restriction is Associated with Functional Delayed Graft Function in Living Donor Kidney Transplantation: A Retrospective Cohort Analysis. J. Clin. Med..

[41] Thölking G., Schütte-Nütgen K., Schmitz J., Rovas A., Dahmen M., Bautz J., Jehn U., Pavenstädt H., Heitplatz B., Van Marck V. (2019). A Low Tacrolimus Concentration/Dose Ratio Increases the Risk for the Development of Acute Calcineurin Inhibitor-Induced Nephrotoxicity. J. Clin. Med..

[42] Douwes R.M., Neto G., Eisenga M., Vinke J.S.J., Borst D., Berg V.D., Berger S.P., Touw D.J., Hak E., Blokzijl H. (2019). Chronic Use of Proton-Pump Inhibitors and Iron Status in Renal Transplant Recipients. J. Clin. Med..

[43] Neuwirt H., Leitner-Lechner I., Kerschbaum J., Ertl M., Pöggsteiner F., Pölt N., Mätzler J., Sprenger-Mähr H., Rudnicki M., Schratzberger P. (2019). Efficacy and Safety of Belatacept Treatment in Renal Allograft Recipients at High Cardiovascular Risk-A Single Center Experience. J. Clin. Med..

[44] Lemerle M., Garnier A.-S., Planchais M., Brilland B., Delneste Y., Subra J.-F., Blanchet O., Blanchard S., Croué A., Duveau A. (2019). CD45RC Expression of Circulating CD8+ T Cells Predicts Acute Allograft Rejection: A Cohort Study of 128 Kidney Transplant Patients. J. Clin. Med..

[45] Sugünes N., Bichmann A., Biernath N., Peters R., Budde K., Liefeldt L., Schlomm T., Friedersdorff F. (2019). Analysis of the Effects of Day-Time vs. Night-Time Surgery on Renal Transplant Patient Outcomes. J. Clin. Med..

[46] Basha J.A., Kiel M., Görlich D., Schütte-Nütgen K., Witten A., Pavenstädt H., Kahl B.C., Dobrindt U., Reuter S. (2019). Phenotypic and Genotypic Characterization of Escherichia coli Causing Urinary Tract Infections in Kidney-Transplanted Patients. J. Clin. Med..

[47] Go J., Park S.C., Yun S.-S., Ku J., Park J., Shim J.-W., Lee H., Kim Y., Moon Y.E., Hong S.H. (2019). Exposure to Hyperchloremia Is Associated with Poor Early Recovery of Kidney Graft Function after Living-Donor Kidney Transplantation: A Propensity Score-Matching Analysis. J. Clin. Med..

[48] Shimada H., Uchida J., Nishide S., Kabei K., Kosoku A., Maeda K., Iwai T., Naganuma T., Takemoto Y., Nakatani T. (2019). Comparison of Glucose Tolerance between Kidney Transplant Recipients and Healthy Controls. J. Clin. Med..

[49] Suarez M.L.G., Thongprayoon C., Mao M.A., Leeaphorn N., Bathini T., Cheungpasitporn W. (2019). Outcomes of Kidney Transplant Patients with Atypical Hemolytic Uremic Syndrome Treated with Eculizumab: A Systematic Review and Meta-Analysis. J. Clin. Med..

[50] Bellini M.I., Charalampidis S., Stratigos I., Dor F., Papalois V. (2019). Dor The Effect of Donors’ Demographic Characteristics in Renal Function Post-Living Kidney Donation. Analysis of a UK Single Centre Cohort. J. Clin. Med..

[51] Attias P., Melica G., Boutboul D., De Castro N., Audard V., Stehlé T., Gaube G., Fourati S., Botterel F., Fihman V. (2019). Epidemiology, Risk Factors, and Outcomes of Opportunistic Infections after Kidney Allograft Transplantation in the Era of Modern Immunosuppression: A Monocentric Cohort Study. J. Clin. Med..

[52] Schütte-Nütgen K., Thölking G., Steinke J., Pavenstädt H., Schmidt R., Suwelack B., Reuter S. (2019). Correction: Fast Tac Metabolizers at Risk-It is Time for a C/D Ratio Calculation. J. Clin. Med..

[53] Chewcharat A., Thongprayoon C., Bathini T., Aeddula N.R., Boonpheng B., Kaewput W., Watthanasuntorn K., Lertjitbanjong P., Sharma K., Torres-Ortiz A. (2019). Incidence and Mortality of Renal Cell Carcinoma after Kidney Transplantation: A Meta-Analysis. J. Clin. Med..

[54] Cheungpasitporn W., Thongprayoon C., Ungprasert P., Wijarnpreecha K., Kaewput W., Leeaphorn N., Bathini T., Chebib F.T., Kroner P. (2019). Subarachnoid Hemorrhage in Hospitalized Renal Transplant Recipients with Autosomal Dominant Polycystic Kidney Disease: A Nationwide Analysis. J. Clin. Med..

[55] Cho Y.H., Hyun H.S., Park E., Moon K.C., Min S.I., Ha J., Ha I.S., Cheong H.I., Ahn Y.H., Kang H.G. (2019). Higher Incidence of BK Virus Nephropathy in Pediatric Kidney Allograft Recipients with Alport Syndrome. J. Clin. Med..

[56] Yepes-Calderón M., Sotomayor C.G., Gomes-Neto A.W., Gans R.O.B., Berger S.P., Rimbach G., Esatbeyoglu T., Rodrigo R., Geleijnse J.M., Navis G. (2019). Plasma Malondialdehyde and Risk of New-Onset Diabetes after Transplantation in Renal Transplant Recipients: A Prospective Cohort Study. J. Clin. Med..

[57] Nieuwenhuijs-Moeke G.J., Pischke S.E., Berger S.P., Sanders J., Pol R.A., Struys M., Ploeg R.J., Leuvenink H.G.D. (2020). Ischemia and Reperfusion Injury in Kidney Transplantation: Relevant Mechanisms in Injury and Repair. J. Clin. Med..

[58] Alcendor D.J. (2019). BK Polyomavirus Virus Glomerular Tropism: Implications for Virus Reactivation from Latency and Amplification during Immunosuppression. J. Clin. Med..

[59] Bellini M.I., Nozdrin M., Yiu J., Papalois V. (2019). Machine Perfusion for Abdominal Organ Preservation: A Systematic Review of Kidney and Liver Human Grafts. J. Clin. Med..

[60] Visser I.J., van der Staaij J.P.T., Muthusamy A., Willicombe M., Lafranca J.A., Dor F. (2019). Timing of Ureteric Stent Removal and Occurrence of Urological Complications after Kidney Transplantation: A Systematic Review and Meta-Analysis. J. Clin. Med..

[61] Thongprayoon C., Acharya P., Aeddula N.R., Torres-Ortiz A., Bathini T., Sharma K., Ungprasert P., Watthanasuntorn K., Suarez M.L.G., Salim S.A. (2019). Effects of denosumab on bone metabolism and bone mineral density in kidney transplant patients: A systematic review and meta-analysis. Arch Osteoporos..

[62] Cheungpasitporn W., Thongprayoon C., Wijarnpreecha K., Mitema D.G., Mao M.A., Nissaisorakarn P., Podboy A., Kittanamongkolchai W., Sakhuja A., Erickson S.B. (2017). Decline in prevalence and risk of helicobacter pylori in kidney transplant recipients: A systematic review and meta-analysis. J. Evid.-Based Med..

[63] Boonpheng B., Thongprayoon C., Bathini T., Sharma K., Mao M.A., Cheungpasitporn W. (2019). Proton pump inhibitors and adverse effects in kidney transplant recipients: A meta-analysis. World J. Transplant..

[64] Thongprayoon C., Chokesuwattanaskul R., Bathini T., Khoury N.J., Sharma K., Ungprasert P., Prasitlumkum N., Aeddula N.R., Watthanasuntorn K., Salim S.A. (2018). Epidemiology and Prognostic Importance of Atrial Fibrillation in Kidney Transplant Recipients: A Meta-Analysis. J. Clin. Med..

[65] Cheungpasitporn W., Thongprayoon C., A Mao M., A Mao S., D’Costa M.R., Kittanamongkolchai W., Kashani K.B. (2017). Contrast-induced acute kidney injury in kidney transplant recipients: A systematic review and meta-analysis. World J. Transplant..

[66] Cheungpasitporn W., Thongprayoon C., A Mao M., Kittanamongkolchai W., Sathick I.J.J., Dhondup T., Erickson S.B. (2016). Incidence of kidney stones in kidney transplant recipients: A systematic review and meta-analysis. World J. Transplant..

[67] Thongprayoon C., Khoury N.J., Bathini T., Aeddula N.R., Boonpheng B., Leeaphorn N., Ungprasert P., Bruminhent J., Lertjitbanjong P., Watthanasuntorn K. (2019). BK polyomavirus genotypes in renal transplant recipients in the United States: A meta-analysis. J. Evid. Based Med..

[68] Schinstock C., Gandhi M., Cheungpasitporn W., Mitema D., Prieto M., Dean P., Cornell L., Cosio F., Stegall M. (2017). Kidney Transplant with Low Levels of DSA or Low Positive B-Flow Crossmatch. Transplant..

[69] Cheungpasitporn W., Kremers W.K., Lorenz E., Amer H., Cosio F.G., Stegall M.D., Gandhi M.J., Schinstock C.A. (2018). De novo donor-specific antibody following BK nephropathy: The incidence and association with antibody-mediated rejection. Clin. Transplant..

[70] Cheungpasitporn W., Thongprayoon C., Mitema D.G., Mao M.A., Sakhuja A., Kittanamongkolchai W., Gonzalez-Suarez M.L., Erickson S.B. (2017). The effect of aspirin on kidney allograft outcomes; a short review to current studies. J. Nephropathol..

[71] Cheungpasitporn W., Thongprayoon C., Harindhanavudhi T., Edmonds P., Erickson S.B. (2016). Hypomagnesemia linked to new-onset diabetes mellitus after kidney transplantation: A systematic review and meta-analysis. Endocr. Res..

[72] Manohar S., Thongprayoon C., Cheungpasitporn W., Markovic S.N., Herrmann S.M. (2019). Systematic Review of the Safety of Immune Checkpoint Inhibitors Among Kidney Transplant Patients. Kidney Int. Rep..

[73] Cheungpasitporn W., Thongprayoon C., Vijayvargiya P., Anthanont P., Erickson S.B. (2016). The Risk for New-Onset Diabetes Mellitus after Kidney Transplantation in Patients with Autosomal Dominant Polycystic Kidney Disease: A Systematic Review and Meta-Analysis. Can. J. Diabetes.

[74] Cheungpasitporn W., Khoury N.J., Thongprayoon C., Craici I.M. (2017). Is Remote Ischemic Conditioning of Benefit to Patients Undergoing Kidney Transplantation?. J. Investig. Surg..

[75] Cheungpasitporn W., Thongprayoon C., Erickson S.B. (2015). Outcomes of living kidney donors with monoclonal gammopathy of undetermined significance. Ren. Fail..

[76] Cheungpasitporn W., Thongprayoon C., Ungprasert P., Erickson S.B. (2015). Outcomes of Living Kidney Donors with Rheumatoid Arthritis. Prog. Transplant..

[77] Thongprayoon C., Kaewput W., Sharma K., Wijarnpreecha K., Leeaphorn N., Ungprasert P., Sakhuja A., Rivera F.H.C., Cheungpasitporn W. (2018). Outcomes of kidney transplantation in patients with hepatitis B virus infection: A systematic review and meta-analysis. World J. Hepatol..

[78] Schinstock C., Cosio F., Cheungpasitporn W., Dadhania D.M., Everly M.J., Samaniego-Picota M.D., Cornell L., Stegall M.D. (2017). The Value of Protocol Biopsies to Identify Patients with De Novo Donor-Specific Antibody at High Risk for Allograft Loss. Arab. Archaeol. Epigr..

[79] Leeaphorn N., Thongprayoon C., Chon W.J., Cummings L.S., Mao M.A., Cheungpasitporn W. (2019). Outcomes of kidney retransplantation after graft loss as a result of BK virus nephropathy in the era of newer immunosuppressant agents. Arab. Archaeol. Epigr..

[80] Chewcharat A., Chang Y.T., Thongprayoon C., Crisafio A., Bathini T., Mao M.A., Cheungpasitporn W. (2020). Efficacy and safety of febuxostat for treatment of asymptomatic hyperuricemia among kidney transplant patients: A meta-analysis of observational studies. Clin. Transplant..

[81] Cheungpasitporn W., Thongprayoon C., Edmonds P., Bruminhent J., Tangdhanakanond K. (2015). The effectiveness and safety of rituximab as induction therapy in ABO-compatible non-sensitized renal transplantation: A systematic review and meta-analysis of randomized controlled trials. Ren. Fail..

[82] Cheungpasitporn W., Thongprayoon C., Mao M.A., Kittanamongkolchai W., Sathick I.J.J., Erickson S.B. (2016). The Effect of Renin-angiotensin System Inhibitors on Kidney Allograft Survival: A Systematic Review and Meta-analysis. N. Am. J. Med. Sci..

[83] Cheungpasitporn W., Thongprayoon C., Ungprasert P., Wijarnpreecha K., Mao M.A., Aeddula N.R., Kaewput W., Bathini T., Kroner P.T. (2020). Hepatitis A hospitalizations among kidney transplant recipients in the United States: Nationwide inpatient sample 2005–2014. Eur. J. Gastroenterol. Hepatol..

[84] Cheungpasitporn W., Chebib F.T., Cornell L.D., Brodin M.L., Nasr S.H., Schinstock C., Stegall M.D., Amer H. (2015). Intravitreal Antivascular Endothelial Growth Factor Therapy May Induce Proteinuria and Antibody Mediated Injury in Renal Allografts. Transplantation.

[85] Farouk S., Zhang Z., Menon M. (2019). Non-HLA donor–recipient mismatches in kidney transplantation—A stone left unturned. Arab. Archaeol. Epigr..

[86] Starzl T.E., Marchioro T.L., Holmes J.H., Hermann G., Brittain R.S., Stonington O.H., Talmage D.W., Waddell W.R. (1964). Renal homografts in patients with major donor-recipient blood group incompatibilities. Surgery.

[87] Sumitran-Holgersson S., Wilczek H.E., Holgersson J., Soderstrom K. (2002). Identification of the nonclassical HLA molecules, mica, as targets for humoral immunity associated with irreversible rejection of kidney allografts1. Transplantation.

[88] Dragun D., Muller D.N., Brasen J.H., Fritsche L., Nieminen-Kelha M., Dechend R., Kintscher U., Rudolph B., Hoebeke J., Eckert D. (2005). Angiotensin II type 1-receptor activating antibodies in renal-allograft rejection. N. Engl. J. Med..

[89] Banasik M., Boratyńska M., Kościelska-Kasprzak K., Kamińska D., Zmonarski S., Mazanowska O., Krajewska M., Bartoszek D., Żabińska M., Myszka-Kozłowska M. (2014). Non-HLA Antibodies: Angiotensin II Type 1 Receptor (Anti-AT1R) and Endothelin-1 Type A Receptor (Anti-ETAR) Are Associated With Renal Allograft Injury and Graft Loss. Transplant. Proc..

[90] Besarani D., Cerundolo L., Smith J.D., Procter J., Barnardo M.C.N., Roberts I.S.D., Friend P.J., Rose M.L., Fuggle S.V. (2014). Role of Anti-Vimentin Antibodies in Renal Transplantation. Transplantation.

[91] Cardinal H., Dieudé M., Brassard N., Qi S., Patey N., Soulez M., Beillevaire D., Echeverry F., Daniel C., Durocher Y. (2013). Antiperlecan Antibodies Are Novel Accelerators of Immune-Mediated Vascular Injury. Arab. Archaeol. Epigr..

[92] Jackson A.M., Sigdel T.K., Delville M., Hsieh S.-C., Dai H., Bagnasco S., Montgomery R.A., Sarwal M.M. (2014). Endothelial Cell Antibodies Associated with Novel Targets and Increased Rejection. J. Am. Soc. Nephrol..

[93] Sablik K.A., Van Groningen M.C.C.-, Damman J., Roelen D.L., Betjes M. (2019). Banff lesions and renal allograft survival in chronic-active antibody mediated rejection. Transplant. Immunol..

[94] Chiu H.-F., Wen M.-C., Wu M.-J., Chen C.-H., Yu T.-M., Chuang Y.-W., Huang S.-T., Tsai S.-F., Lo Y.-C., Ho H.-C. (2020). Treatment of chronic active antibody-mediated rejection in renal transplant recipients-a single center retrospective study. BMC Nephrol..

[95] Nair P., Gheith O., Al-Otaibi T., Mostafa M., Rida S., Sobhy I., Halim M.A., Mahmoud T., Abdul-Hameed M., Maher A. (2019). Management of Chronic Active Antibody-Mediated Rejection in Renal Transplant Recipients: Single-Center Experience. Exp. Clin. Transplant..

[96] Choi J., Aubert O., Vo A., Loupy A., Haas M., Puliyanda D., Kim I., Louie S., Kang A., Peng A. (2017). Assessment of Tocilizumab (Anti-Interleukin-6 Receptor Monoclonal) as a Potential Treatment for Chronic Antibody-Mediated Rejection and Transplant Glomerulopathy in HLA-Sensitized Renal Allograft Recipients. Arab. Archaeol. Epigr..

[97] Vo A.A., Aubert O., Haas M., Huang E., Zhang X., Choi J., Peng A., Najjar R., Sethi S., Ammerman N. (2019). Clinical Relevance of Posttransplant DSAs in Patients Receiving Desensitization for HLA-incompatible Kidney Transplantation. Transplantation.

[98] Pottebaum A.A., Venkatachalam K., Liu C., Brennan D.C., Murad H., Malone A.F., Alhamad T. (2020). Efficacy and Safety of Tocilizumab in the Treatment of Acute Active Antibody-mediated Rejection in Kidney Transplant Recipients. Transplant. Direct.

[99] Eskandary F., Duerr M., Budde K., Doberer K., Reindl-Schwaighofer R., Waiser J., Wahrmann M., Regele H., Spittler A., Lachmann N. (2019). Clazakizumab in late antibody-mediated rejection: Study protocol of a randomized controlled pilot trial. Trials.

[100] Jordan S.C., Ammerman N., Choi J., Huang E., Peng A., Sethi S., Najjar R., Toyoda M., Lim K., Louie S. (2019). Novel Therapeutic Approaches to Allosensitization and Antibody-mediated Rejection. Transplantation.

[101] Jordan S.C., Choi J., Aubert O., Haas M., Loupy A., Huang E., Peng A., Kim I., Louie S., Ammerman N. (2018). A phase I/II, double-blind, placebo-controlled study assessing safety and efficacy of C1 esterase inhibitor for prevention of delayed graft function in deceased donor kidney transplant recipients. Arab. Archaeol. Epigr..

[102] Montgomery R.A., Orandi B.J., Racusen L., Jackson A.M., Garonzik-Wang J.M., Shah T., Woodle E.S., Sommerer C., Fitts D., Rockich K. (2016). Plasma-Derived C1 Esterase Inhibitor for Acute Antibody Mediated Rejection Following Kidney Transplantation: Results of a Randomized, Double-Blind, Placebo-Controlled Pilot Study. Arab. Archaeol. Epigr..

[103] Böhmig G.A., Eskandary F., Doberer K., Halloran P.F. (2019). The therapeutic challenge of late antibody-mediated kidney allograft rejection. Transplant. Int..

[104] Rangaswami J., Mathew R.O., Parasuraman R., Tantisattamo E., Lubetzky M., Rao S., Yaqub M.S., Birdwell K.A., Bennett W., Dalal P. (2019). Cardiovascular disease in the kidney transplant recipient: Epidemiology, diagnosis and management strategies. Nephrol. Dial. Transplant..

[105] Ponticelli C., Diekmann F., Graziani G. (2011). Hypertension in kidney transplant recipients. Transplant. Int..

[106] Cannon R., Jones C.M., Hughes M.G., Eng M., Marvin M.R. (2013). The Impact of Recipient Obesity on Outcomes After Renal Transplantation. Ann. Surg..

[107] Holdaas H., Fellström B., Jardine A. (2005). Clinical Practice Guidelines for Managing Dyslipidemias in Kidney Transplant Patients: Lessons to be Learnt From the Assessment of LescolR in Renal Transplantation (ALERT) Trial. Arab. Archaeol. Epigr..

[108] Kasiske B.L. (2005). Clinical practice guidelines for managing dyslipidemias in kidney transplant patients. Am. J. Transplant..

[109] (2009). Special Issue: KDIGO Clinical Practice Guideline for the Care of Kidney Transplant Recipients. Arab. Archaeol. Epigr..

[110] Mallamaci F., D’Arrigo G., Tripepi R., Leonardis D., Porto G., Testa A., Elhafeez S.A., Mafrica A., Versace M.C., Provenzano P.F. (2018). Office, standardized and 24-h ambulatory blood pressure and renal function loss in renal transplant patients. J. Hypertens..

[111] Kasiske B.L., Anjum S., Shah R., Skogen J., Kandaswamy C., Danielson B., O’Shaughnessy E.A., Dahl D.C., Silkensen J.R., Sahadevan M. (2004). Hypertension after kidney transplantation. Am. J. Kidney Dis..

[112] Whelton P.K., Carey R.M., Aronow W.S., Casey N.E., Collins K.J., Himmelfarb C.D., DePalma S.M., Gidding S., Jamerson K.A., Jones D.W. (2017). 2017 ACC/AHA/AAPA/ABC/ACPM/AGS/APhA/ASH/ASPC/NMA/PCNA Guideline for the Prevention, Detection, Evaluation, and Management of High Blood Pressure in Adults: Executive Summary: A Report of the American College of Cardiology/American Heart Association Task Force on Clinical Practice Guidelines. Hypertension.

[113] Cosio F.G., Kudva Y., Van Der Velde M., Larson T.S., Textor S.C., Griffin M.D., Stegall M.D. (2005). New onset hyperglycemia and diabetes are associated with increased cardiovascular risk after kidney transplantation. Kidney Int..

[114] Weinrauch L., Claggett B., Liu J., Finn P.V., Weir M.R., E Weiner D., A D’Elia J. (2018). Smoking and outcomes in kidney transplant recipients: A post hoc survival analysis of the FAVORIT trial. Int. J. Nephrol. Renov. Dis..

[115] Holdaas H., Fellström B., Jardine A., Nyberg G., Grönhagen-Riska C., Madsen S., Neumayer H.-H., Cole E., Maes B., Ambühl P. (2005). Beneficial effect of early initiation of lipid-lowering therapy following renal transplantation. Nephrol. Dial. Transplant..

[116] Nicoletto B.B., Fonseca N.K.O., Manfro R.C., Gonçalves L.F.S., Leitao C., Souza G.C. (2014). Effects of Obesity on Kidney Transplantation Outcomes. Transplantation.

[117] Weiner D.E., A Carpenter M., Levey A.S., Ivanova A., Cole E.H., Hunsicker L., Kasiske B.L., Kim S.J., Kusek J.W., Bostom A.G. (2012). Kidney function and risk of cardiovascular disease and mortality in kidney transplant recipients: The FAVORIT trial. Arab. Archaeol. Epigr..

[118] Fernández-Fresnedo G., Escallada R., Rodrigo E., De Francisco A.L.M., Cotorruelo J.G., De Castro S.S., Zubimendi J.A., Ruiz J.C., Arias M. (2002). The risk of cardiovascular disease associated with proteinuria in renal transplant patients. Transplantation.

[119] Hiremath S., A Fergusson D., Fergusson N., Bennett A., Knoll G.A. (2017). Renin-Angiotensin System Blockade and Long-term Clinical Outcomes in Kidney Transplant Recipients: A Meta-analysis of Randomized Controlled Trials. Am. J. Kidney Dis..

[120] Paoletti E., Bellino D., Signori A., Pieracci L., Marsano L., Russo R., Massarino F., Ravera M., Fontana I., Carta A. (2015). Regression of asymptomatic cardiomyopathy and clinical outcome of renal transplant recipients: A long-term prospective cohort study. Nephrol. Dial. Transplant..

[121] Ketteler M., Block G.A., Evenepoel P., Fukagawa M., Herzog C.A., McCann L., Moe S.M., Shroff R., Tonelli M., Toussaint N.D. (2017). Executive summary of the 2017 KDIGO Chronic Kidney Disease–Mineral and Bone Disorder (CKD-MBD) Guideline Update: What’s changed and why it matters. Kidney Int..

[122] Rigatto C. (2003). Electrocardiographic Left Ventricular Hypertrophy in Renal Transplant Recipients: Prognostic Value and Impact of Blood Pressure and Anemia. J. Am. Soc. Nephrol..

[123] Israni A.K., Snyder J.J., Skeans M.A., Peng Y., MacLean J.R., Weinhandl E.D., Kasiske B.L. (2010). For the PORT Investigators Predicting Coronary Heart Disease after Kidney Transplantation: Patient Outcomes in Renal Transplantation (PORT) Study. Arab. Archaeol. Epigr..

[124] Kasiske B.L., A Chakkera H., Roel J. (2000). Explained and unexplained ischemic heart disease risk after renal transplantation. J. Am. Soc. Nephrol..

[125] Gaston R.S., Basadonna G., Cosio F.G., Davis C.L., Kasiske B.L., Larsen J., Leichtman A.B., Delmonico F. (2004). Transplantation in the diabetic patient with advanced chronic kidney disease: A task force report. Am. J. Kidney Dis..

[126] Balla A., Chobanian M. (2009). New-onset diabetes after transplantation: A review of recent literature. Curr. Opin. Organ. Transplant..

[127] Munagala M.R., Phancao A. (2016). Managing Cardiovascular Risk in the Post Solid Organ Transplant Recipient. Med. Clin. N. Am..

[128] Beshyah S.A., Beshyah A.S., Beshyah W.S., Yaghi S. (2018). Use of SGLT2 Inhibitors in Diabetic Renal Transplant Recipients: A Mixed Method Exploratory Exercise. Int. J. Diabetes Metab..

[129] Conte C., Secchi A. (2018). Post-transplantation diabetes in kidney transplant recipients: An update on management and prevention. Acta Diabetol..

[130] Campistol J.M. (2008). Minimizing the Risk of Posttransplant Malignancy. Transplant. Proc..

[131] Briggs J.D. (2001). Causes of death after renal transplantation. Nephrol. Dial. Transplant..

[132] Rama I., Grinyó J.M. (2010). Malignancy after renal transplantation: The role of immunosuppression. Nat. Rev. Nephrol..

[133] Wang Y., Lan G.B., Peng F.H., Xie X.B. (2018). Cancer risks in recipients of renal transplants: A meta-analysis of cohort studies. Oncotarget.

[134] White S.L., Rawlinson W., Boan P., Sheppeard V., Wong G., Waller K., Opdam H., Kaldor J., Fink M., Verran D. (2019). Infectious Disease Transmission in Solid Organ Transplantation: Donor Evaluation, Recipient Risk, and Outcomes of Transmission. Transplant. Direct.

[135] Howard R.J., Patton P.R., Reed A., Hemming A.W., Van Der Werf W.J., Pfaff W.W., Srinivas T.R., Scornik J.C. (2002). The changing causes of graft loss and death after kidney transplantation. Transplantation.

[136] Ramaswamy K., Madariaga H.M., Thomas B.S., Lerma E. (2020). Kidney transplantation for the primary care provider. Disease.

[137] Chesdacha S., Thongprayoon C., Bruminhent J., Cheungpasitporn W. (2017). Efficacy and adverse effects of cidofovir for treatment of BK virus infection in kidney transplant recipients. J. Nephropharmacol..

[138] Vanichanan J., Udomkarnjananun S., Avihingsanon Y., Jutivorakool K. (2018). Common viral infections in kidney transplant recipients. Kidney Res. Clin. Pract..

[139] Hilbrands L.B. (2020). Latest developments in living kidney donation. Curr. Opin. Organ. Transplant..

[140] A Montgomery R., Gentry S., Marks W.H., Warren D.S., Hiller J., Houp J., A Zachary A., Melancon J.K., Maley W.R., Rabb H. (2006). Domino paired kidney donation: A strategy to make best use of live non-directed donation. Lancet.

[141] Lee L.-Y., Pham T.A., Melcher M.L. (2019). Living Kidney Donation: Strategies to Increase the Donor Pool. Surg. Clin. N. Am..

[142] Warren D.S., Montgomery R.A. (2010). Incompatible kidney transplantation: Lessons from a decade of desensitization and paired kidney exchange. Immunol. Res..

[143] De Klerk M., Kal-van Gestel J.A., van de Wetering J., Kho M.L., Middel-de Sterke S., Betjes M.G.H., Zuidema W.C., Roelen D., Glorie K., Roodnat J.I. (2020). Creating Options for Difficult-to-match Kidney Transplant Candidates. Transplantation.

[144] Ross L.F., Rodrigue J.R., Veatch R.M. (2017). Ethical and Logistical Issues Raised by the Advanced Donation Program “Pay It Forward” Scheme. J. Med. Philos..

[145] Doshi M.D., Ortigosa-Goggins M., Garg A.X., Li L., Poggio E.D., Winkler C.A., Kopp J.B. (2018). APOL1 Genotype and Renal Function of Black Living Donors. J. Am. Soc. Nephrol..

[146] Thongprayoon C., Cheungpasitporn W. (2017). Persistent hyperparathyroidism after kidney transplantation; updates on the risk factors and its complications. J. Parathyr. Dis..

[147] Yoo K.D., Noh J., Lee H., Kim D.K., Lim C.S., Kim Y.H., Lee J.P., Kim G., Kim Y.S. (2017). A Machine Learning Approach Using Survival Statistics to Predict Graft Survival in Kidney Transplant Recipients: A Multicenter Cohort Study. Sci. Rep..

[148] Mark E., Goldsman D., Gurbaxani B., Keskinocak P., Sokol J. (2019). Using machine learning and an ensemble of methods to predict kidney transplant survival. PLoS ONE.

[149] Bae S., Massie A.B., Thomas A.G., Bahn G., Luo X., Jackson K.R., Ottmann S.E., Brennan D.C., Desai N.M., Coresh J. (2018). Who can tolerate a marginal kidney? Predicting survival after deceased donor kidney transplant by donor-recipient combination. Arab. Archaeol. Epigr..

[150] Atallah D.M., Badawy M., El-Sayed A., Ghoneim M.A. (2019). Predicting kidney transplantation outcome based on hybrid feature selection and KNN classifier. Multimed. Tools Appl..

[151] Nematollahi M., Akbari R., Nikeghbalian S., Salehnasab C. (2017). Classification Models to Predict Survival of Kidney Transplant Recipients Using Two Intelligent Techniques of Data Mining and Logistic Regression. Int. J. Organ. Transplant. Med..

[152] Tapak L., Hamidi O., Amini P., Poorolajal J. (2017). Prediction of Kidney Graft Rejection Using Artificial Neural Network. Healthc. Inform. Res..

[153] Shahmoradi L., Langarizadeh M., Pourmand G., Fard Z.A., Borhani A. (2016). Comparing Three Data Mining Methods to Predict Kidney Transplant Survival. Acta Inform. Med..

[154] Luck M., Sylvain T., Cardinal H., Lodi A., Bengio Y. (2017). Deep Learning for Patient-Specific Kidney Graft Survival Analysis. arXiv.

[155] Topuz K., Zengul F.D., Dag A., Almehmi A., Yildirim M.B. (2018). Predicting graft survival among kidney transplant recipients: A Bayesian decision support model. Decis. Support Syst..

[156] Thongprayoon C., Kaewput W., Kovvuru K., Hansrivijit P., Kanduri S.R., Bathini T., Chewcharat A., Leeaphorn N., Gonzalez-Suarez M.L., Cheungpasitporn W. (2020). Promises of Big Data and Artificial Intelligence in Nephrology and Transplantation. J. Clin. Med..

[157] Forbes R.C., Rybacki D.B., Johnson T.B., Hannah-Gillis A., Shaffer D., Hale D.A. (2018). A Cost Comparison for Telehealth Utilization in the Kidney Transplant Waitlist Evaluation Process. Transplantation.

[158] Andrew N., Barraclough K.A., Long K., Fazio T.N., Holt S., Kanhutu K., Hughes P.D. (2018). Telehealth model of care for routine follow up of renal transplant recipients in a tertiary centre: A case study. J. Telemed. Telecare.

